# Long-Term Coffee Consumption Is Associated with Decreased Incidence of New-Onset Hypertension: A Dose–Response Meta-Analysis

**DOI:** 10.3390/nu9080890

**Published:** 2017-08-17

**Authors:** Giuseppe Grosso, Agnieszka Micek, Justyna Godos, Andrzej Pajak, Salvatore Sciacca, Maira Bes-Rastrollo, Fabio Galvano, Miguel A. Martinez-Gonzalez

**Affiliations:** 1 NNEdPro Global Centre for Nutrition and Health, St John’s Innovation Centre, Cambridge CB4 0WS, UK; 2 Integrated Cancer Registry of Catania-Messina-Siracusa-Enna, Azienda Ospedaliero-Universitaria Policlinico-Vittorio Emanuele, 95123 Catania, Italy; justyna.godos@student.uj.edu.pl (J.G.); sciacca@unict.it (S.S.); 3 Department of Epidemiology and Population Studies, Jagiellonian University Medical College, 31-008 Krakow, Poland; agnieszka.micek@uj.edu.pl (A.M.); andrzej.pajak@uj.ed.pl (A.P.); 4 Department of Preventive Medicine & Public Health, School of Medicine, University of Navarra, 31008 Pamplona, Spain; mbes@unav.es (M.B.-R.); mamartinez@unav.es (M.A.M.-G.); 5 IDISNA (Navarra’s Health Research Institute, 31008 Pamplona, Spain; 6 CIBERobn, Instituto de Salud Carlos III, 28029 Madrid, Spain; 7 Department of Biomedical and Biotechnological Sciences, University of Catania, 95124 Catania, Italy; fgalvano@unict.it

**Keywords:** coffee, hypertension, risk, cohort, smoking, meta-analysis

## Abstract

Objective: To perform a dose–response meta-analysis of prospective cohort studies investigating the association between long-term coffee intake and risk of hypertension. Methods: An online systematic search of studies published up to November 2016 was performed. Linear and non-linear dose–response meta-analyses were conducted; potential evidence of heterogeneity, publication bias, and confounding effect of selected variables were investigated through sensitivity and meta-regression analyses. Results: Seven cohorts including 205,349 individuals and 44,120 cases of hypertension were included. In the non-linear analysis, there was a 9% significant decreased risk of hypertension per seven cups of coffee a day, while, in the linear dose–response association, there was a 1% decreased risk of hypertension for each additional cup of coffee per day. Among subgroups, there were significant inverse associations for females, caffeinated coffee, and studies conducted in the US with longer follow-up. Analysis of potential confounders revealed that smoking-related variables weakened the strength of association between coffee consumption and risk of hypertension. Conclusions: Increased coffee consumption is associated with a modest decrease in risk of hypertension in prospective cohort studies. Smoking status is a potential effect modifier on the association between coffee consumption and risk of hypertension.

## 1. Introduction

Coffee has been the focus of major attention due to its global consumption and impact on health [1]. Historically, coffee consumption was considered to have detrimental effects on health, particularly its contribution to high blood pressure [2]. However, coffee contains many other bioactive compounds, such as polyphenols, furans, pyrroles, and maltol, all of which have recently been hypothesized to have potential beneficial effects on human health [3,4]. Meta-analyses of prospective cohort studies show a J-shaped dose–response relationship between coffee consumption and decreased risk of cardiovascular disease (CVD), including coronary heart disease and stroke [5], and mortality [6] suggesting that moderate consumption is key for its beneficial effects. Studies exploring acute events most likely triggered by coffee-dependent increase in blood pressure showed no substantial increase in mortality due to myocardial infarction [7] and atrial fibrillation [8]. Apart from cardiovascular health, coffee consumption has been hypothesized to affect a number of other conditions, including metabolic status [6] and risk of late-life cognitive impairment [9,10].

Overall, the benefits of coffee on human health seem evident; however, the association between coffee consumption and in particular risk of hypertension remains open for debate. Results from meta-analyses of randomized controlled trials testing the effects of caffeine intake and decaffeinated coffee on blood pressure were null and showed no increase in blood pressure [11,12]; however, these studies simply evaluated the effect of the caffeine in coffee and not of coffee itself. Due to the relatively short-term follow-up, previous studies mostly emphasized the acute effects of coffee intake rather than its long-term effects on blood pressure. Two previous meta-analyses of prospective cohort studies [13,14] reported non-significant increased risk of hypertension associated with higher intake of coffee. However, those meta-analyses only included a few studies reporting dose–response analysis and did not correct for the number of individuals/cases/person-years, and potential confounding factors/effect modifiers, ultimately providing limited-quality evidence. In light of these limitations and the availability of newly published cohort studies, this study aims to conduct a dose–response meta-analysis of prospective cohort studies investigating coffee consumption and the risk of hypertension.

## 2. Materials and Methods

The design, analysis, and reporting of this study is compliant with the Preferred Reporting Items for Systematic Reviews and Meta-Analyses (PRISMA) guidelines (Table S1).

### 2.1. Study Selection

A systematic search of PubMed (http://www.ncbi.nlm.nih.gov/pubmed/) and EMBASE (http://www.embase.com/) databases was conducted for studies up to November 2016 using the following search strategy: (“coffee” (MeSH Terms) OR “coffee” (All Fields)) AND (“hypertension” (MeSH Terms) OR “hypertension” (All Fields)) AND (cohort (All Fields) OR (“longitudinal studies” (MeSH Terms) OR (“longitudinal” (All Fields) AND “studies” (All Fields)) OR “longitudinal studies” (All Fields) OR “prospective” (All Fields) OR cases (All Fields). The inclusion criteria consisted of studies that: (i) are prospective; (ii) evaluated the association of coffee intake and the risk of hypertension in individuals without hypertension at baseline; (iii) assessed and reported hazard ratios (HRs), risk ratios (RRs), or odds ratios (ORs) and 95% CI for hypertension; and (iv) assessed and reported a defined amount of coffee consumption (i.e., cups per day) for each category of exposure. Reference lists of the included studies were also searched for any additional study not previously identified. For papers that studied the same cohort, only the study that included the entire cohort or had the longest follow-up was included.

### 2.2. Data Extraction

Data were extracted by two independent investigators (G.G. and A.M.) using a standardized extraction form. Discrepancies on the included studies were discussed and resolved by consensus. The following information was collected: (i) first author name; (ii) year of publication; (iii) study cohort name and country; (iv) total number, sex, and age (mean or range) of participants; (v) follow-up period; (vi) distributions of cases and person-years, HRs/RRs/ORs and 95% CIs for all categories of exposure; (vii) coffee intake range for each category of exposure; (viii) adjusted covariates; (ix) prevalence of smokers, diabetic subjects, and participants with low physical activity for each category of exposure; and (x) baseline mean/median systolic and diastolic blood pressure, age, body mass index (BMI), and daily intake of sodium and potassium for each category of coffee intake.

### 2.3. Study Quality Assessment

The quality of each study was assessed according to the Newcastle-Ottawa Quality Assessment Scale [15] composed of 3 variables: selection (4 points), comparability (2 points), and outcome (3 points) resulting in a total score of 9 points (9 representing the highest quality). Studies scoring 7–9 points, 3–6 points, and 0–3 points were identified as high, moderate, and low quality, respectively.

### 2.4. Statistical Analysis

In this meta-analysis, ORs and HRs referring to new onset incident cases of hypertension were deemed equivalent to risk ratios (RRs) [16]. When coffee consumption was illustrated by ranges of intake, the midpoint of the range was used. When the highest category was open-ended, we assumed that the width of the upper category was the same as the adjacent category. When the lowest category was open-ended, we set the lower boundary to zero. Two-stage random-effects dose–response meta-analysis was performed to examine linear and non-linear relationship between coffee intake and risk of developing hypertension during follow-up.

First, generalized least-squares (GLS) were used to calculate study-specific coefficients across categories of coffee intake accounting for the correlation within each set of RRs [17,18]. Non-linear dose–response analysis was modeled using restricted cubic splines with 3 knots at fixed percentiles (25%, 50%, and 75%) of the distribution [19]. Coefficients estimated within each study were combined by performing random-effects meta-analysis. DerSimonian and Laird’s method was used for linear dose–response meta-analysis and, in non-linear dose–response meta-analysis, the multivariate extension of the method of moments was used to estimate the RRs. We tested whether the 2 regression coefficients were simultaneously equal to zero and calculated a *p*-value for non-linearity by testing whether the coefficient of the second spline was equal to zero. A sensitivity analysis by excluding one study at the time was conducted to determine the stability and robustness of the results.

To test for potential confounders, subgroup analyses by sex, type of coffee, and geographical area were performed. To further investigate whether other unmeasured potential confounders should be included in the interpretation of the results, we investigated the distribution of systolic blood pressure, diastolic blood pressure, age, BMI, daily intake of sodium and potassium, percentage of smokers, participants with low physical activity and those with type-2 diabetes across categories of coffee consumption. For this purpose, a separate two-stage bivariate meta-analysis was performed for each confounder variable to determine its association with coffee intake [20]. First, linear regression coefficients (slope and intercept) between coffee intake and the potential confounders above were estimated. Second, we used GLS to synthesize these intercepts and slope coefficients, accounting for the corresponding variance-covariance matrices [21]. To determine the significance of the proposed confounders on the association of coffee consumption and risk of hypertension, a meta-regression analysis was conducted. Specifically, we used the joint slope coefficient of the association as the moderator in the meta-regression analysis. Percentage of smokers was also used as moderator.

Publication bias was assessed using Egger’s regression test while the Cochran *Q*-test tested for statistical heterogeneity (statistical significance is defined as a *p* value less than 0.10) and quantified through the multivariate generalization of the *I*^2^ statistic (no, low, medium, and high heterogeneity were defined by *I*^2^ values <25%, 25–50%, 50–75%, and >75%, respectively). All analyses were performed on R, software version 3.0.3, dosresmeta and mvmeta packages (Development Core Team, Vienna, Austria).

## 3. Results

### 3.1. Study Characteristics

**Figure 1:**
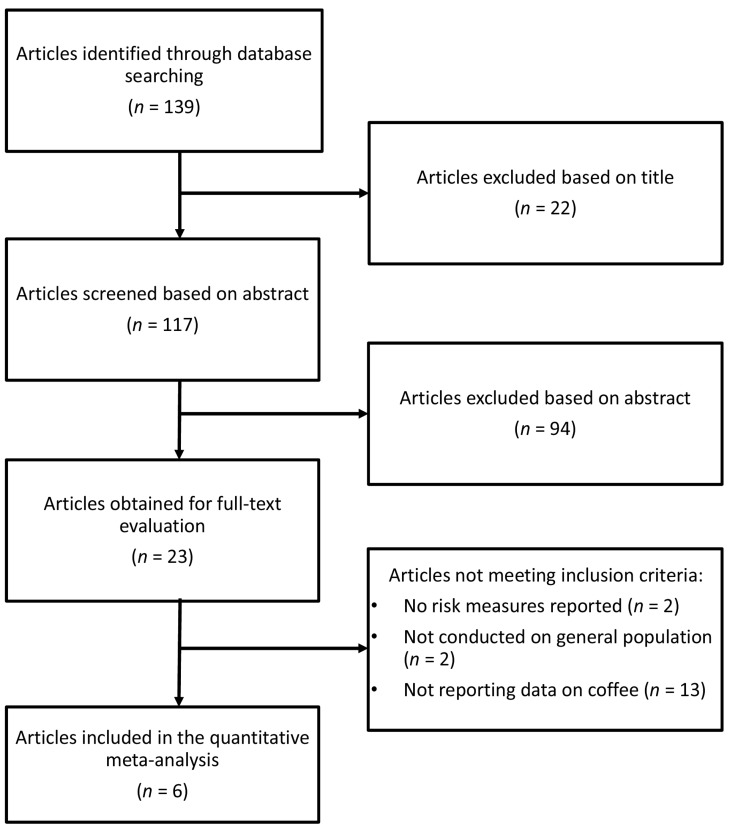
Flowchart of study selection for inclusion in the meta-analysis.

The selection process of studies included in the meta-analysis is illustrated in Figure 1. Out of the 139 studies screened, six studies [22,23,24,25,26,27] involving seven cohorts, 205,349 individuals, and 44,120 cases of hypertension were included (Table 1). The sample of three studies included general population [24,25,26], registered US nurses (2 cohorts) [23], post-menopausal women [27], and post-graduate students [22]. Four cohorts were based in the US [22,23,27] and three in Europe [24,25,26]. Sex-specific (both male and female) risk estimates were provided by three studies [24,25,26], while two papers [23,27] studied only females and one only males [22]. Follow-up periods ranged an average of 3–33 years. All studies adjusted for variables that affect the risk of hypertension, including age, gender, BMI, and smoking status. Overall, the quality of all included studies was high (data not shown).

### 3.2. Coffee Consumption and Risk of Hypertension

**Figure 2:**
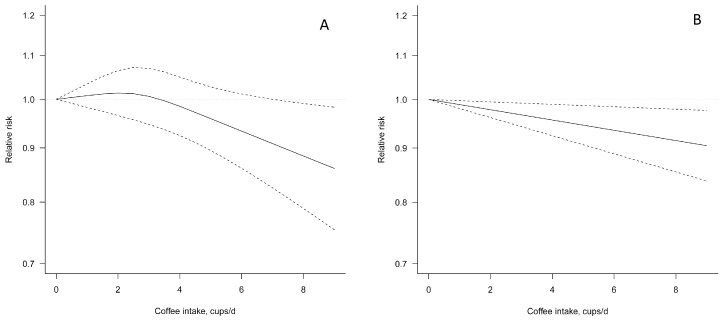
Dose–response association between coffee consumption and risk of hypertension: (**A**) non-linear association; and (**B**) linear association. Solid lines represent risk ratio, dashed lines represent 95% confidence intervals.

**Figure 3:**
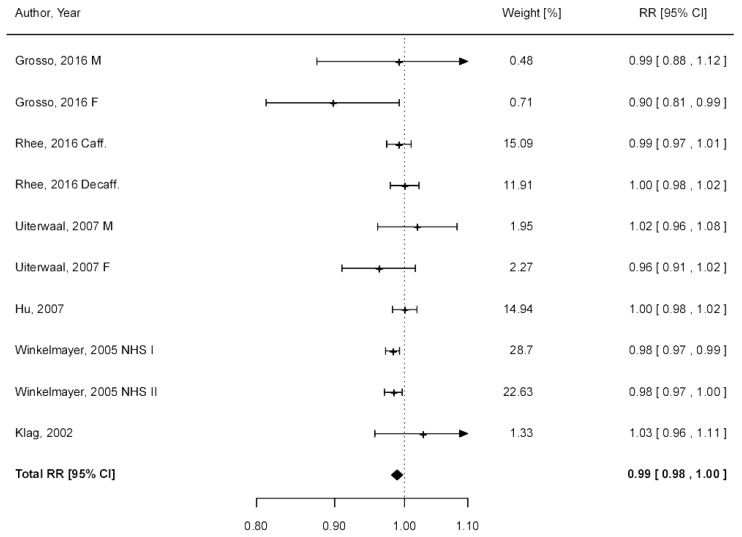
Forest plot illustrating risk of hypertension due to increase intake of one-cup of coffee per day in cohort studies.

The dose–response regression in Figure 2A illustrates a non-significant decrease in risk of hypertension with up to six cups/day and a 9% significant decrease in risk observed for seven cups/day (RR = 0.91, 95% CI: 0.83–1.00), with moderate evidence of heterogeneity (*I*^2^ = 51%, *P_heterogeneity_* = 0.004) and no publication bias (*P_Egger_* = 0.755) (Table 2). After exclusion of one study [24], the association between coffee consumption and risk of hypertension became significant with no evidence of heterogeneity and publication bias (Table S2). When linear dose–response regression (test for non-linearity resulted not significant) was modeled, similar risk estimates resulted (Figure 2B), where there was a 1% decreased risk of hypertension with each additional cup of coffee per day (RR = 0.99, 95% CI: 0.98, 1.00; *I*^2^ = 21%, *P_heterogeneity_* = 0.241; Figure 3). Stratified analyses by sex, geographical area, type of coffee consumed, and length of follow-up were limited by the small number of datasets; however, significant inverse associations were observed in studies conducted in the US, and those that studied only females, caffeinated coffee, and had longer follow-up (Table 2).

### 3.3. Potential Confounding Factors

**Figure 4:**
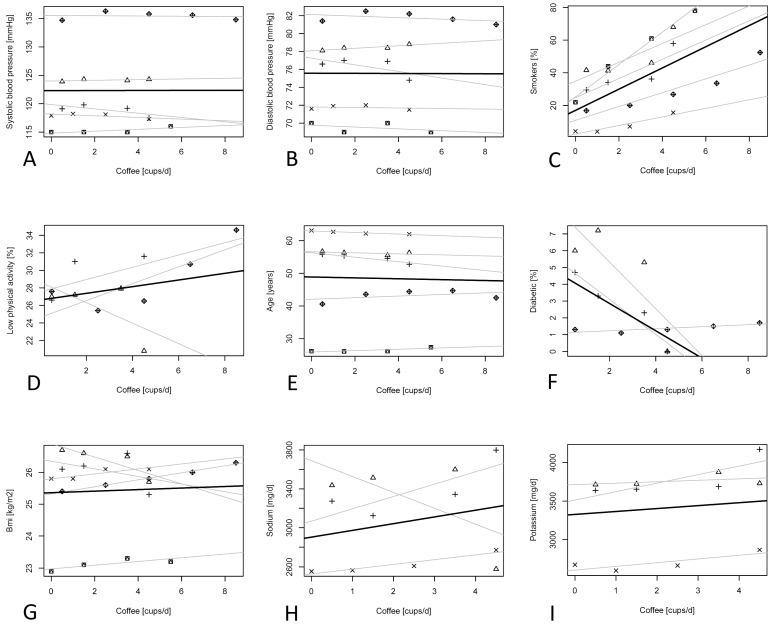
Scatter plots illustrating the relationship between coffee consumption (in cups per day) and baseline characteristics in the prospective cohorts: (**A**) systolic blood pressure; (**B**) diastolic blood pressure; (**C**) percentage of smokers; (**D**) percentage of low physically active individuals; (**E**) age; (**F**) percentage of diabetic individuals; (**G**) body mass index; (**H**) sodium intake; and (**I**) potassium intake. Symbols represent different cohorts; light lines represent linear regression coefficients of individual studies; bold lines represent summary estimates of average increase of each variable with increase in coffee intake. Cups/d: cups/day.

The distribution of systolic and diastolic blood pressure and percentage of smokers across categories of coffee consumption was explored in four studies where a significant association was evident only with diastolic blood pressure (Figure 4). The prevalence of smokers in the no-coffee consumption category was 16.8%, which increased by about 6.4% for each additional cup of coffee consumed per day (Table 3).

**Figure 5:**
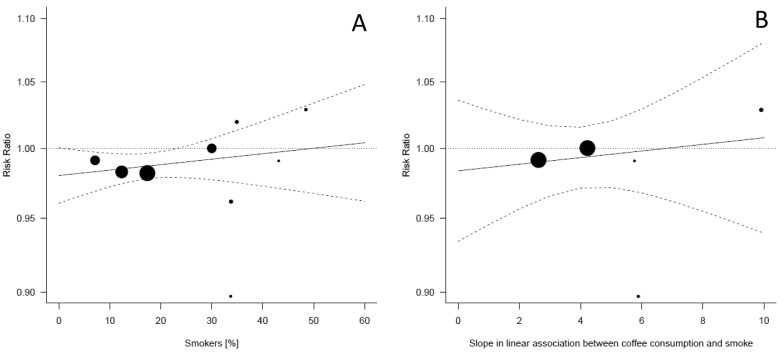
Risk of hypertension for linear increase of coffee consumption and selected smoking-related moderators: (**A**) total prevalence of smokers in the included cohorts; and (**B**) slope coefficients of the association between coffee intake and percentage of smokers in each category of exposure and random effects meta-regression. The area of each circle is inversely proportional to the variance of the log risk ratio estimate; continuous lines represent average risk ratios; and dashed lines represent corresponding 95% confidence intervals.

Figure 5 illustrates two meta-regression models to test the confounding role of smoking status on the association of coffee consumption and risk of hypertension, both of which resulted in insignificant associations. With prevalence of smokers as the moderator (Figure 5A), studies with lower prevalence showed that coffee consumption was associated with decreased risk of hypertension, while in studies with higher prevalence, coffee consumption was associated with increased risk. With slope coefficients as the moderator (Figure 5B), studies with no change in prevalence of smokers across categories of coffee consumption (lower slope values) showed that coffee consumption was associated with decreased risk of hypertension, while, in studies where increased coffee consumption was linearly associated with higher prevalence of smokers (higher slope values), coffee consumption was associated with increased risk of hypertension.

## 4. Discussion

This meta-analysis illustrated a linear association between increasing intake of coffee consumption and decreased risk of hypertension in six prospective cohort studies. However, when smoking status was included as a moderator in the meta-regression analyses, the association was weakened, suggesting that smoking may be an effect modifier. Compared to previous meta-analyses [13,14], this study included a larger sample (39,078 additional individuals and 8257 additional cases), dose–response analyses, and tested for potential confounders. The comparison of different categories of exposure (i.e., the variation of coffee intake across studies), may have biased the pooled risk estimates of previous meta-analyses of cohort studies. Differences in the results from this study compared to those from meta-analyses of randomized controlled trials [11,12] may be due to the potential long-term effect of coffee consumption captured only in cohort studies.

From the subgroup analyses, this study showed significant results in studies (or datasets) that only included women, were conducted in the US, and had a longer follow-up. Significant results seen in women and not in men could be attributed to increased prevalence of smoking in males accounting for 30% of all mortality in males 50–70 years [28]. To account for geographical differences in the results, the number of participants in US cohorts was three times more than the European cohorts. Thus, it can be inferred the number of individuals in the European cohorts was not large enough to obtain the expected effect size. This is in fact in line with the results obtained, as risk estimates in both subgroups (US and European cohorts) were similar but the analysis of the European cohorts had larger confidence intervals. Furthermore, significant results seen in studies with longer follow-up seems intuitive since studies with longer follow-up more likely capture potential cases of chronic non-communicable disease.

Apart from those above, other reasons may account for the differences observed between subgroups (especially those between geographical areas), including variations in preferred coffee constituents, variations in brewing and in the actual amount of coffee contained in “a cup” across regions, and differences in population genetics. There is evidence that genetic factors related to target receptors and CYP1A2, such as the genetic polymorphism rs762551, also known as −136 C>A CYP1A2, encoding the CYP1A2*1F allele of the CYP1A2 gene, leads to slow metabolism of caffeine [29,30]. Carriers of the CYP1A2*1F allele have been demonstrated to be at higher risk of nonfatal myocardial infarction following intake of caffeinated coffee [31]. Similarly, the risk of sustained hypertension has been associated with CYP1A2*1F polymorphisms in a cohort of stage I hypertensive individuals [32]. Due to the variability of genetic polymorphisms of CYP1A2 enzyme, also related to ethnicity, it is plausible that there exist such genetic polymorphisms in the examined cohorts that result in the heterogeneity of our results, especially those found across geographical regions.

Another finding of this study was the potential effect modification of sodium intake on risk of hypertension associated with coffee consumption. Despite insufficient data for further examining this issue, three studies showed direct association between sodium intake and coffee consumption where increased intake of one cup of coffee per day was associated with 38 mg/day additional intake of sodium. It is known that increased sodium intake may be associated with increased fluid retention, but does not increase urine volume excretion, thus leading to a rise in body weight and blood pressure [33]. A comprehensive systematic review exploring data from 93 studies including more than 600 empirically-derived dietary patterns reported that coffee was part of a “healthy” dietary pattern in 4 studies and part of an “unhealthy” dietary pattern in 11 studies, suggesting that coffee drinkers may have, at least in part, unhealthier dietary choices, which may include higher sodium intake [34]. However, it is unclear whether the reported increase in sodium intake may have clinical relevance and more studies are needed to better explore this potential association.

The biological evidence supporting the association between coffee consumption and decreased risk of hypertension discounting caffeine intake has been investigated only during the last decade. The coffee content in phenolic compounds has been considered the main factor responsible for the beneficial effects on blood pressure mentioned previously [2]. The most studied compounds are chlorogenic acids, including metabolites ferulic acid, caffeic acid and quinic acid, which have all been reported to exert anti-hypertensive effects in experimental studies [35]. The main mechanisms of action rely on the anti-oxidant activity of chlorogenic acid, through its inhibition of the NAD(P)H oxidase expression and activity and by directly scavenging for free radicals [36]. Moreover, chlorogenic acid has been reported to stimulate nitric oxide production by the endothelial-dependent pathway, suggesting that vascular integrity, in particular, intact endothelium, is essential for chlorogenic acids to have a blood pressure lowering effect [37,38].

This meta-analysis has some limitations that should be addressed. The main issue in interpreting our findings depends on the potential confounders (smoking status, sodium intake) and genetic polymorphisms that are not explored in existing cohorts. We attempted an in-depth analysis on the role of smoking status, suggesting that it poses as an effect modifier. No data on genetic polymorphisms and only limited data on sodium intake were available and, hence, we were not able to analyze the potential effect modification of these variables, which may have weakened the observed results. Nevertheless, the large number of individuals included, the lack of evidence on heterogeneity, and the observed significant inverse association observed for the main effect variables all support the robustness of the findings. Another limitation in the papers included in this study is the lack of information on the type of coffee used (e.g., boiled, filtered, etc.) and on the actual amount of coffee used in one cup. The type of coffee consumed by US cohorts could be different from those consumed by European cohorts, which contribute to the heterogeneous findings. Similarly, the amount of coffee added in a cup can vary across countries. One cup of coffee could refer to 150 mL or 300 mL which is another source of heterogeneity in these results. However, a previous meta-analysis that adjusted for the varying measurements of coffee showed identical results to the unadjusted measures on the outcome investigated [6].

Considering that the results from this meta-analysis may not account for the genetic variations contributing to the slow metabolism of caffeine, overall current evidence suggest that usual coffee consumption in the long-term is not a risk factor for developing hypertension in existing cohort studies. Rather results show an inverse association of increase coffee consumption and the risk of developing hypertension. Due to lack of long-term randomized controlled trials, results from cohort studies represent the most reliable available evidence when studying long-term effects of food on future health.

To date, our study provides a robust overview of the highest level of evidence that currently exists on the association of coffee consumption and risk of hypertension. Nevertheless, more cohort studies are needed to provide more in-depth and long-term evidence of this association. Additional factors such as smoking status, sodium intake and genetic polymorphisms need to be addressed in future studies to allow for stratified sub-group analyses. Further evidence on decaffeinated coffee could also provide information on the role of caffeine in such long-term outcomes.

## Figures and Tables

**Table 1:** Main characteristics of prospective cohort studies investigating the association of coffee consumption and risk of hypertension.

Author, Year	Name of Cohort (Years of Recruitment), Country	Cohort Size	No. of Cases	Gender	Exposure Variables	Follow-Up Duration	Adjusted Variables
Grosso, 2016 (21)	HAPIEE Cohort Study (2002–2008), Poland	2725	1735	MF	Caffeinated and decaffeinated coffee	5 y	Age, gender, education, occupation, BMI, alcohol consumption, smoking status, physical activity level, past history of CVD and diabetes at baseline, cholesterol therapy at baseline, total energy intake, vitamin supplement use, oral contraceptive use, and intake of sodium and potassium.
Rhee, 2016 (22)	WHI Observational Study, (1993), US	29,985	5566	F	Caffeinated coffee	3 y	Age, baseline blood pressure, BMI, physical activity, hormone replacement therapy, alcohol consumption, smoking status, total calorie intake, and intake of sodium, magnesium, calcium, potassium, and phosphorus as time-varying covariates.
Uiterwaal, 2007 (20)	DCS (1987–2002), The Netherlands	6368	956	MF	Caffeinated and decaffeinated coffee	11 y	Baseline age, sex, height and weight, smoking, alcohol intake, tea intake, education level, occupational status, and total energy intake.
Hu, 2007 (19)	Four independent surveys, (1982–2002), Finland	24,710	2505	MF	Caffeinated and decaffeinated coffee	13.2 y	Year, education, leisure-time physical activity, smoking status, alcohol consumption, tea consumption, frequency of vegetable, fruit, sausage, and bread consumption, BMI, history of diabetes and total cholesterol, and baseline systolic blood pressure.
Winkelmayer, 2005 (18)	NHS I (1976–2002), US	53,175	19,541	F	Caffeinated coffee	12 y	Age, BMI, alcohol consumption, family history of hypertension, physical activity, and smoking status, and intake of other beverages.
Winkelmayer, 2005 (18)	NHS II (1989–2003), US	87,369	13,536	F	Caffeinated coffee	12 y	Age, BMI, alcohol consumption, family history of hypertension, oral contraceptive use, physical activity, and smoking status, and intake of other beverages.
Klag, 2002 (17)	JHPS (1947–1995), US	1017	281	M	Caffeinated coffee	33 y	Parental history of hypertension, time-dependent number of cigarettes smoked, alcohol intake, physical activity, BMI.

DCS: Doetinchem Cohort Study; HAPIEE: Health, Alcohol and Psychosocial factors In Eastern Europe; JHPS: John Hopkins Precursors Study; NHS: Nurses’ Health Study; WHI: Women’s Health Initiative Observational Study; y: year.

**Table 2:** Dose–response meta-analysis of coffee consumption and risk of hypertension, stratified by selected variables.

	No. of Datasets (No. of Studies)	Coffee Intake (Cups/Day)								*I*^2^	*P_heterogeneity_*
		0	1	2	3	4	5	6	7		
All											
Non-linear	10 (6)	Ref.	1.01 (0.98–1.03)	1.01 (0.97–1.06)	1.01 (0.95–1.07)	0.98 (0.92–1.05)	0.96 (0.89–1.03)	0.93 (0.86–1.01)	0.91 (0.83–1.00)	51%	0.004
Linear	10 (6)	Ref.	0.99 (0.98–1.00)	0.98 (0.96–0.99)	0.97 (0.94–0.99)	0.96 (0.92–0.99)	0.95 (0.91–0.99)	0.94 (0.89–0.98)	0.92 (0.87–0.98)	21%	0.241
Males											
Non-linear	3 (3)	Ref.	1.09 (0.94–1.27)	1.19 (0.89–1.58)	1.24 (0.88–1.76)	1.23 (0.89–1.69)	1.19 (0.9–1.59)	1.16 (0.86–1.55)	1.12 (0.79–1.59)	0%	0.458
Linear	3 (3)	Ref.	1.02 (0.98–1.06)	1.04 (0.95–1.13)	1.06 (0.93–1.21)	1.08 (0.91–1.28)	1.1 (0.89–1.36)	1.12 (0.86–1.45)	1.14 (0.84–1.55)	0%	0.458
Females											
Non-linear	4 (4)	Ref.	1.00 (0.97–1.03)	1.00 (0.94–1.05)	0.98 (0.92–1.04)	0.95 (0.91–0.99)	0.92 (0.88–0.95)	0.88 (0.84–0.94)	0.85 (0.78–0.93)	43%	0.059
Linear	6 (4)	Ref.	0.99 (0.98–0.99)	0.97 (0.95–0.99)	0.96 (0.93–0.98)	0.94 (0.91–0.98)	0.93 (0.89–0.97)	0.92 (0.87–0.97)	0.90 (0.85–0.96)	26%	0.232
Caffeinated coffee											
Non-linear	4 (3)	Ref.	1.00 (0.97–1.02)	0.99 (0.95–1.04)	0.98 (0.93–1.03)	0.95 (0.92–0.99)	0.92 (0.89–0.96)	0.9 (0.84–0.96)	0.87 (0.79–0.96)	44%	0.095
Linear	4 (3)	Ref.	0.98 (0.98–0.99)	0.97 (0.95–0.98)	0.95 (0.93–0.98)	0.94 (0.91–0.97)	0.92 (0.89–0.96)	0.91 (0.87–0.95)	0.9 (0.85–0.94)	0%	0.529
Europe											
Non-linear	5 (3)	Ref.	1.02 (0.9–1.16)	1.04 (0.81–1.33)	1.06 (0.76–1.48)	1.08 (0.8–1.47)	1.12 (0.92–1.35)	1.15 (0.98–1.35)	1.19 (0.91–1.55)	54%	0.025
Linear	5 (3)	Ref.	0.99 (0.96–1.02)	0.97 (0.91–1.04)	0.96 (0.87–1.05)	0.95 (0.83–1.07)	0.93 (0.79–1.09)	0.92 (0.76–1.11)	0.91 (0.72–1.13)	38%	0.166
US											
Non-linear	3 (3)	Ref.	1 (0.98–1.02)	1 (0.96–1.04)	0.98 (0.94–1.03)	0.96 (0.92–0.99)	0.92 (0.89–0.96)	0.89 (0.84–0.94)	0.86 (0.79–0.93)	36	0.130
Linear	5 (3)	Ref.	0.99 (0.98–0.99)	0.97 (0.96–0.99)	0.96 (0.94–0.98)	0.95 (0.92–0.97)	0.93 (0.9–0.97)	0.92 (0.88–0.96)	0.91 (0.86–0.95)	1%	0.398
Follow up >10 years											
Non-linear	6 (4)	Ref.	1.02 (0.99–1.04)	1.03 (0.98–1.09)	1.02 (0.96–1.10)	0.99 (0.91–1.08)	0.95 (0.85–1.05)	0.91 (0.79–1.03)	0.87 (0.74–1.01)	57%	0.01
Linear	6 (4)	Ref.	0.99 (0.98–1.00)	0.97 (0.96–0.99)	0.96 (0.93–0.99)	0.95 (0.91–0.99)	0.94 (0.89–0.98)	0.93 (0.87–0.98)	0.91 (0.85–0.98)	21%	0.277
Follow up <10 years											
Non-linear	4 (2)	Ref.	0.97 (0.91–1.04)	0.95 (0.85–1.07)	0.94 (0.83–1.07)	0.94 (0.83–1.07)	0.95 (0.83–1.08)	0.95 (0.82–1.1)	0.95 (0.80–1.12)	43%	0.101
Linear	4 (2)	Ref.	0.99 (0.97–1.01)	0.98 (0.94–1.02)	0.97 (0.92–1.04)	0.97 (0.89–1.05)	0.96 (0.86–1.06)	0.95 (0.84–1.07)	0.94 (0.82–1.09)	31%	0.225

**Table 3:** Summary of the associations between coffee consumption and selected variables.

	No. of Datasets (No. of Studies)	Intercept (95% CI)	Slope per 1 Cup/Day (95% CI)	*p* for Slope
SBP (mmHg)	5 (4)	122.3 (115.2, 129.3)	0.01 (−0.12, 0.13)	0.9345
DBP (mmHg)	5 (4)	75.6 (71.2, 79.8)	−0.01 (−0.14, 0.12)	0.9131
Smokers (%)	5 (4)	16.9 (7.2, 26.7)	6.49 (3.77, 9.22)	<0.001
BMI	5 (4)	25.4 (24.1, 26.6)	0.02 (−0.08, 0.13)	0.6461
Age (year)	5 (4)	48.9 (35.9, 61.8)	−0.13 (−0.42, 0.15)	0.3565
Sodium (mg/day)	3 (2)	2903.2 (2480.9, 3325.6)	69.2 (37.0, 101.4)	<0.001
Potassium (mg/day)	3 (2)	3326.6 (2613.5, 4039.7)	38.0 (3.2, 72.8)	0.0326
Low PA (%)	3 (2)	26.6 (23.8, 29.4)	0.38 (−0.83, 1.59)	0.5391
Diabetic (%)	3 (2)	4.48 (0.51, 8.45)	−0.81 (−1.84, 0.22)	0.1218

BMI: body mass index; DBP: diastolic blood pressure; PA: physical activity; SBP: systolic blood pressure.

## Supplementary Materials

The following are available online at  www.mdpi.com/2072-6643/9/8/890/s1.
